# Efficient Synthesis of 2-Ethylhexanoic Acid via *N*-Hydroxyphthalimide Catalyzed Oxidation of 2-Ethylhexanal with Oxygen

**DOI:** 10.3390/ma16175778

**Published:** 2023-08-23

**Authors:** Łukasz Czieszowic, Beata Orlińska, Dawid Lisicki, Ewa Pankalla

**Affiliations:** 1 Grupa Azoty Zakłady Azotowe–Kędzierzyn-S.A., Mostowa 30A, 47-220 Kędzierzyn-Koźle, Poland; ewa.pankalla@grupaazoty.com; 2 Department of Chemical Organic Technology and Petrochemistry and PhD School, Silesian University of Technology, Akademicka 2A, 44-100 Gliwice, Poland; dawid.lisicki@polsl.pl

**Keywords:** oxidation, 2-ethylhexanoic acid, *N*-hydroxyphthalimide, aldehyde

## Abstract

An efficient method for the synthesis of 2-ethylhexanoic acid has been reported. The method involves the 2-ethylhexanal oxidation using oxygen or air in the presence of *N*-hydroxyphthalimide in *iso*butanol as a solvent under mild conditions. A high selectivity of >99% for 2-ethylhexanoic acid was achieved. The influence of catalyst amount, solvent type and quantity, temperature, and reaction time on the product composition was studied. The developed method is in line with the global trends aimed at developing green oxidation processes as well as having potential for implementation in industry due to its high selectivity, cost-effective oxidizing agent, and mild reaction conditions. The use of isobutanol as a solvent is of crucial importance providing an opportunity for potential producers of 2-EHAL from butanal to employ the less valuable alcohol.

## 1. Introduction

2-Ethylhexanoic acid (2-EHA) and its derivatives are widely used in the chemical industry, including production of alkyd resins, plasticizers, stabilizers of polyvinyl chloride, lubricants, detergents, flotation agents, and corrosion inhibitors [1]. 

**Figure 1:**
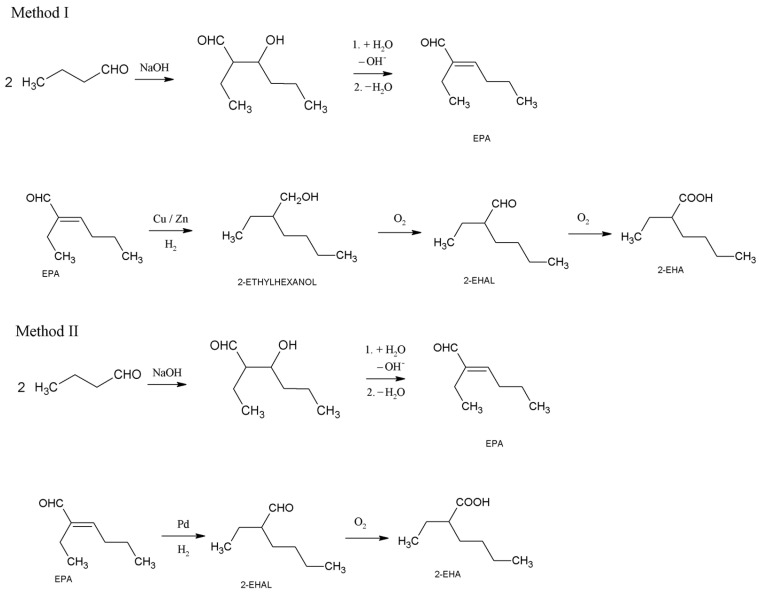
Scheme of obtaining 2-EHA from n-butanal.

There are two primary industrial pathways for 2-EHA synthesis, both starting from butanal, which is a product of propylene hydroformylation (Figure 1). In method (I), 2-ethylhexanol is obtained through butanal aldolization and subsequent hydrogenation. This 2-ethylhexanol is then oxidized to 2-ethylhexanal (2-EHAL), which is further oxidized to 2-EHA. In method (II), an unsaturated aldehyde, 2-ethylhex-2-enal (EPA), is obtained through aldol condensation of butanal, followed by dehydration. Selective hydrogenation of an EPA yields 2-EHAL, which is then oxidized to 2-EHA [1]. Notably, both methods involve the oxidation of 2-EHAL. 

In industry, the exothermic process of 2-EHAL oxidation to 2-EHA (approx. 250 to 300 kJ/mol [1]), which occurs according to a radical chain mechanism [2], is typically carried out in the liquid phase using air [3,4,5], or oxygen [6,7,8]. Various catalysts have been employed, including transition metals, alkaline earth metals, hydroxides, or salts, and combinations of both transition metals and alkaline metal salts [9,10,11,12]. Attempts have also been made to carry out the process in gaseous phase; however, only a mixture of heptane, 3-heptanone, and 3-heptyl formate was obtained in this process [13].

The authors of the paper [14] studied the oxidation of 2-EHAL with air and observed that the conversion of 2-EHAL increased as the temperature rose. The content of aldehyde in the post-reaction mixture was 76% and 26% at 30 °C and 83 °C, respectively. However, the acid 2-EHA content in the products was 18% and 50% at the above-mentioned temperatures. The main byproduct was the ester 3-heptyl formate (6% at 30 °C and 18% at 83 °C). Increasing the molar ratio of oxygen to aldehyde from 0.625 to 10.0 resulted in a higher yield of 2-EHA, increasing from 52% to 67% as well as an increase in ester yield from 19% to 23% [14]. 

Gliński and Kijeński obtained an 80% yield of 2-EHA through oxidation with oxygen at 40 °C, utilizing Mn(II) 2-ethylhexanoate as a catalyst [10]. Lehtinen et al. utilized Mn(II) acetate as the catalyst and octanoic acid as a solvent for 2-EHAL oxidation with oxygen, resulting in 83% yield of the acid [15]. By employing Fe(II), Ni(II), or Co(II) complexes as catalysts, oxygen as the oxidizing agent, and dichloroethane as solvent at room temperature, a 70% yield of 2-EHA was obtained [16]. Ko et al. achieved 84% yield of the acid by oxidizing 2-EHAL with oxygen using KOH as a catalyst in a continuous stirred tank reactor at 50 °C and under pressure of 0.8 MPa [17]. Furthermore, when EHAL was oxidized using oxygen in the presence of Mn(II) 2-ethylhexanoate and sodium 2-ethylhexanoate at room temperature and pressure of 0.5 or 0.75 MPa, a 97–98% yield of 2-EHA was obtained [18,19]. 

The utilization of hydrogen peroxide as an oxidant in 2-EHAL oxidation has also been reported [20]. The authors recommended the use of aqueous solutions of hydrogen peroxide with a concentration of 3% to 30%. A yield of 65% of 2-EHA was achieved when 30% aqueous solution of hydrogen peroxide and a phase transfer catalyst, specifically a quaternary ammonium salt [CH_3_(C_8_H_17_)_3_N]HSO_4_, were applied in a two-phase system at 90 °C for 2 h. However, the use of hydrogen peroxide on an industrial scale may be limited due to its higher price compared to air or oxygen. 

In non-catalytic oxidation processes of 2-EHAL using air or oxygen at a temperature of 82 °C, lower yields of the acid were obtained, respectively, 66 and 50% [21]. Shapiro et al. achieved comparable yields of 2-EHA by oxidizing 2-EHAL in an aqueous suspension using air and oxygen. They extended the reaction time with air by ten hours. A yield of 88% was obtained after 2 h of oxygen oxidation, and when air was used, the yield was 86% after 12 h of reaction [22]. By conducting the 2-EHAL oxidation process in octane as a solvent, with oxygen under pressure of 0.3 MPa and a temperature of 40 °C, 81% of 2-EHA was obtained [23]. 

To enhance the economic and ecological aspects of oxidation processes with industrial importance, novel catalysts have been investigated. Recently, the use of *N*-hydroxyphthalimide (NHPI) as an active organocatalyst for free radical processes [24], including the oxidation reaction of various aldehydes to their corresponding carboxylic acids, has been reported [25]. As a model reaction, the cyclohexanal oxidation was carried out with oxygen at a low temperature of 30 °C for 3 h in various solvents such as acetonitrile, methanol, toluene, water, dioxane, and n-butanol. The authors observed that the highest yields of acid were achieved when using acetonitrile, while cyclohexanal did not undergo oxidation in both alcohols, methanol and n-butanol [25]. Under the optimized conditions, a high yield of 90% for 2-EHA was obtained in MeCN as the solvent. 

Patent authors [26,27] obtained 2-EHA by means of specific chemical reactions. Moiseevich et al. describe a method for obtaining acid from the waste from the production of *N*-2-ethylhexyl-*N’*-phenyl-*N*-phenylenediamine. The authors of the patent alkylated *N*-aminodiphenylamine with a solution of potassium 2-ethylhexanoate. Then, they added water to the mixture and extracted several times with an aliphatic solvent. 2-Ethylhexanoic acid was distilled from the organic layer under reduced pressure with a >99% yield. In the patent [27], the authors used the reaction of 1-amino-2-bromo-3-ethylheptene with dimethyl fumarate. In the process conducted for 3 h at 48 °C, 2-EHA was obtained with 96% yield. The acid was separated from the post-reaction mixture by seven-fold extraction with a solution of 1,3-propanediamine and a nine-fold extraction with propylene glycol methyl ether. The described method allows only limited amounts of 2-EHA to be obtained. For industrial process, new solutions are being sought to improve the economic efficiency while meeting very stringent environmental protection requirements. 

Herein, an environmentally friendly technology of 2-EHAL oxidation to 2-EHA is reported. The developed method uses oxygen as the oxidant and NHPI as the catalyst. It is in line with the current global trends aimed at developing green oxidation processes that use environmentally friendly oxidizing agents. We propose that this method holds potential for implementation in the industry due to its high selectivity, cost-effective oxidizing agent, and mild reaction conditions. Furthermore, the use of isobutanol as the solvent is of crucial importance. The hydroformylation of propylene results in a mixture of both valuable butanal and a smaller amount of less valuable isobutanal. Both aldehydes can be subsequently hydrogenated to respective alcohols. Therefore, this presents an opportunity for potential producers of 2-EHAL from butanal (Figure 1) to utilize the less valuable isobutanol. The potential of employing isobutanol as a solvent in aldehyde’s oxidation with oxygen in the presence of NHPI has been reported for the first time. Previous attempts to use methanol or n-butanol in this reaction did not yield positive outcomes [25].

## 2. Materials and Methods

### 2.1. Materials

*N*-hydroxyphthalimide (Sigma-Aldrich, St. Louis, MO, USA, 97%), acetonitrile (Supelco, Bellefonte, PA, USA, 99.9%), isobutanol (Grupa Azoty ZAK S.A., Kedzierzyn-Kozle, Poland, 99.7%), n-butanol (Grupa Azoty ZAK S.A. 99.8%), 2-ethylhexanol (Grupa Azoty ZAK S.A. 99.7%), heptane (Supelco 99.5%), decane (Sigma-Aldrich 99%), toluene (Chempur, Piekary Slaskie, Poland, 99.5%), acetic acid (Chempur 99.5%), methanol (Chempur 99.8%), and 2-ethylhexanal (Sigma-Aldrich 96%).

### 2.2. 2-EHAL Oxidation Reaction with Oxygen or Air

**Figure 2:**
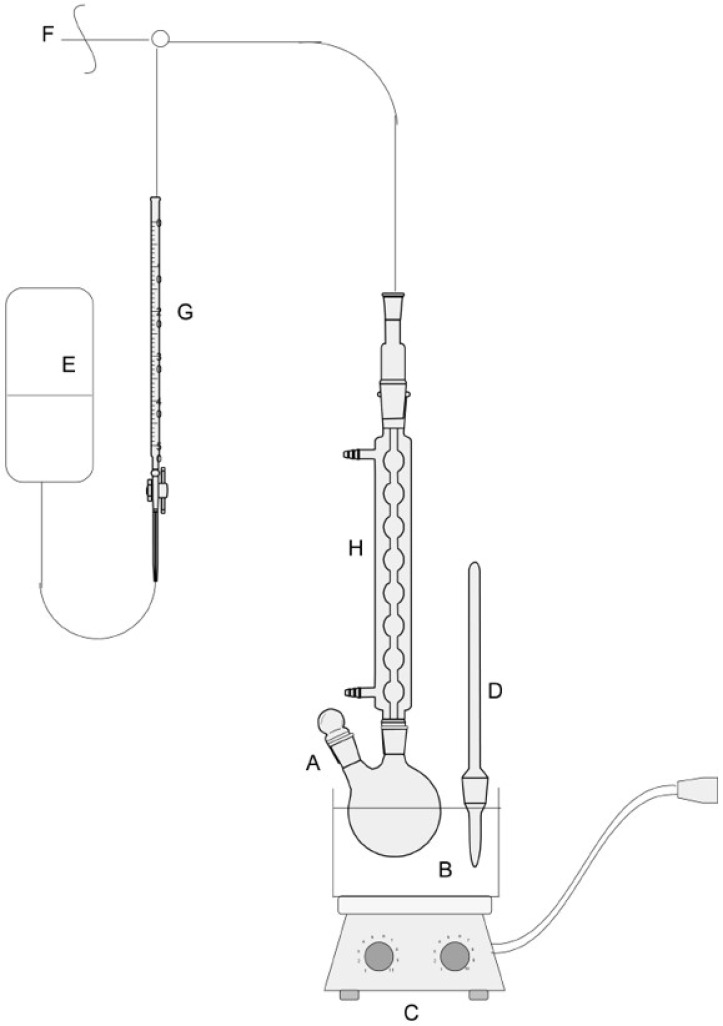
Gasometric apparatus: (A)—two-neck flask, (B)—oil bath, (C)—magnetic stirrer, (D)—thermometer, (E)—water tank, (F)—connection to oxygen cylinder, (G)—measuring burette, (H)—reflux condenser.

The oxidation processes of 2-EHAL with oxygen were carried out using a gasometric apparatus, as depicted in Figure 2. The 2-EHAL, catalysts, and solvent were introduced into a 10 cm^3^ two-necked flask. The reaction flask was connected to a gas burette filled with oxygen at atmospheric pressure. The oxygen uptake was monitored during the oxidation process with an accuracy of 0.1 cm^3^. The conversion of 2-EHAL was calculated based on amount of 2-EHAL in the reaction mixture before and after reaction determined using GC analysis. The selectivity of 2-EHA and byproducts were determined by means of GC analysis. 

### 2.3. Analytical Methods

The composition of the reaction products was analyzed using an Agilent 8890 gas chromatograph equipped with a FID detector, DB-WAXetr column (50 m × 0.32 mm × 1 μm), and automatic sample dispenser. The analysis was carried out using helium as a carrier gas. Dispenser temperature: 250 °C; detector temperature: 270 °C; division: 20:1; injection volume 1 μL; air 400 mL/min.; nitrogen 25 mL/min.; hydrogen 30 mL/min.; oven temperature program: 50 °C, 3 °C/min. to 65 °C, 5 °C/min to 120 °C, 15 °C/min to 200 °C, 200 °C for 15 min. Each sample was analyzed twice and the concentration of the substance was calculated from pre-prepared calibration curves. Samples for analysis were taken directly from the post-reaction mixture, no prior preparation was required prior to GC analysis. The composition of the product was additionally confirmed by the gas chromatograph with mass spectrometry (GC-MS) performed on the Agilent 8890 gas chromatograph equipped with an automatic sample dispenser, DB-5ms column (30 m × 0.25 mm × 0.25 μm, helium 2 mL/min), coupled to the Agilent 5977B GC/MSD (EI 70 eV) mass spectrometer using the NIST mass spectra library.

## 3. Results

In the drive to develop a more sustainable and environmentally friendly method of 2-EHA synthesis with industrial potential, NHPI was selected as the organocatalyst for the oxidation of 2-EHAL using oxygen or air under atmospheric pressure. Due to the limited solubility of NHPI in both the aldehyde and obtained acid, the use of a polar solvent was needed to achieve a homogeneous reaction mixture. The influence of different solvent types and quantities, catalyst amount, temperatures, and reaction time on the conversion of 2-EHAL and the selectivity of 2-EHA were examined. The composition of the resulting mixtures was determined by means of gas chromatography analysis. Apart from 2-EHA, several byproducts were identified in the reaction mixture, including: heptane, 3-heptanone (3H=O), 3-heptanol (3H-OL), and 3-heptyl formate (3HFE).

### 3.1. Effect of Solvent Type

The limited solubility of NHPI in 2-EHAL was demonstrated through solubility tests. Hence, research was conducted to evaluate the effect of different solvents on the studied reaction. Solvents commonly available in the plant producing 2-EHAL, namely iso-butanol (i-BuOH), n-butanol, and 2-ethylhexanol, were used. Additionally, reactions were carried out in acetonitrile (MeCN), heptane, decane, toluene (PhCH_3_), acetic acid (AcOH), and methanol (MeOH) for comparison purposes. The conversion of the raw material, selectivity towards the main product 2-EHA, and byproducts are shown in Table 1.

**Figure 3:**
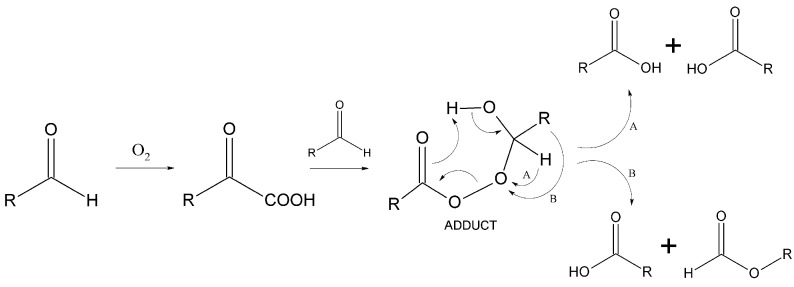
Oxidation of aldehyde to the corresponding acid via reactions of adduct.

It was observed that the use of both polar solvents, such as MeCN or AcOH, and non-polar solvents, such as heptane, decane, and toluene, resulted in high conversions of 2-EHAL (≥99%). However, the 2-EHA selectivity was found to be unsatisfactory, ranging between 47 and 69%. Additionally, it was noted that the share of 3HFE reaction formation was significantly higher in non-polar systems. This suggests that the oxidation mechanism (Figure 3) in non-polar systems favors the migration of the alkyl group (pathway B) rather than hydrogen (pathway A). As a result, the adduct decomposes to the carboxylic acid and formate [15,18,28,29]. The formation of 3HFE in non-polar systems in the amount of several to several dozen percent (Table 1, entry 3–5), aligns with the results obtained by Lehtinen, Nevalainen, and Brunow [29,30]. The oxidation of 2-EHAL did not occur when methanol was used as a solvent, which aligns with the results obtained by Dai, Qu, and Kang in their pioneering use of NHPI for this reaction [19].

Surprisingly, encouraging results were obtained when using the so-called OXO alcohols, namely i-BuOH, n-butanol, and 2-ethylhexanol, as solvents. Although the conversion of 2-EHAL was lower compared to other solvents used (43–55%), the selectivities towards 2-EHA were remarkably high (>90%). Moreover, from a technological standpoint, it is easier to recover and reuse the unreacted raw material 2-EHAL, than to remove various impurities from the final product. Furthermore, it was confirmed that the esterification reaction between 2-EHA and i-BuOH is limited under elevated temperature of studied reaction. Therefore, i-BuOH was chosen for further investigation.

### 3.2. Effect of the Amount of i-BuOH

The effect of the amount of i-BuOH on the oxidation of 2-EHAL in the presence of NHPI with oxygen was determined. The resulting conversion of 2-EHAL, as well as selectivity of the acid and byproducts are depicted in Table 2.

The study revealed that conducting the process using i-BuOH in the amount of 2 cm^3^, corresponding to a concentration of 1 mol/dm^3^, resulted in the highest selectivity towards the acid. Although further reducing the amount of i-BuOH would have been economically advantageous, it might have been insufficient for solubilizing NHPI.

### 3.3. Effect of the Amount of Catalyst

The effect of the amount of NHPI on the oxidation reaction of 2-EHAL at 30 °C using oxygen in i-BuOH was investigated. The results are depicted in Table 3.

It was observed that reducing the NHPI amount below 6 mol% adversely affected the aldehyde conversion, while increasing the catalyst amount to 8 mol% had minimal impact on the conversion decrease. The selectivity remained consistently high (>94%) in all studied reactions using from 2 to 8 mol% of NHPI. In contrast, the selectivity of the non-catalytic reaction was significantly lower. This indicates the key role of the NHPI in the 2-EHA formation. Probably, NHPI participates not only in the formation of the peracid from the aldehyde, as proposed by the authors of [25], but also promotes the peracid decomposition, generating phthalimido-*N*-oxyl radical (PINO) and 2-ethylhexanoyloxyl radical, which is subsequently transformed into 2-EHA. This aligns with the paper regarding the NHPI-catalyzed oxidation of benzaldehyde to benzoic acid [31].

Fine particles of undissolved NHPI were observed in the pre-oxidation mixture for all systems. Additionally, systems containing 6 and 8 mol% of NHPI showed the presence of sediment of undissolved NHPI in the post-reaction mixture. Considering the potential problem of sediment formation in large-volume installations, it was decided to proceed with further research using the addition of NHPI in the amount of 5 mol%.

For comparison, a non-catalytic oxidation reaction of 2-EHAL with oxygen at 30 °C was performed for three hours using MeCN as a solvent. These conditions yielded 95% aldehyde conversion and 38% selectivity towards acid. Subsequently, the oxidation process was conducted in the presence of 5 mol% of NHPI in MeCN, under the same, aforementioned conditions. As a result, the 2-EHAL conversion increased to 99.5% and 2-EHA selectivity to 47%.

### 3.4. Influence of Temperature and Reaction Time

Table 4 presents the results of the studies on the effect of temperature and reaction time on the oxidation of 2-EHAL with oxygen. 

It was observed that increasing the temperature from 30 to 60 °C resulted in an increase in 2-EHAL conversion from 59.0 to >99%. However, this temperature increase also led to a decrease in selectivity to 2-EHA, which was unfavorable.

**Figure 4:**
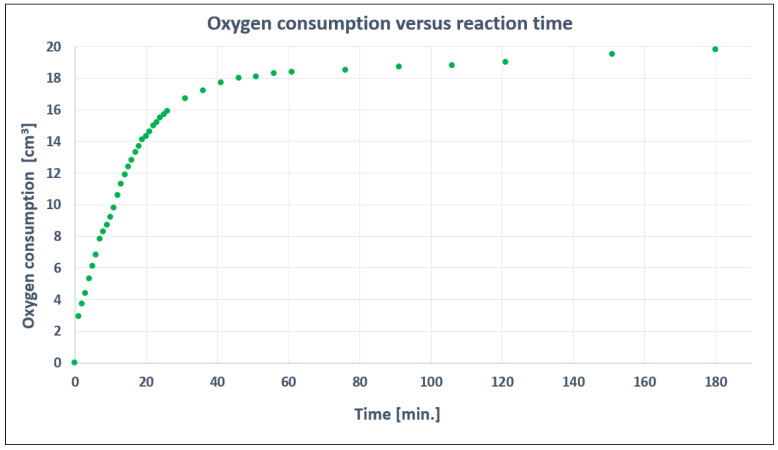
Oxygen consumption versus reaction time.

Extending the reaction time beyond 3 h is not justified as the rate of the 2-EHAL oxidation reaction is low, as demonstrated in Figure 4 which shows the oxygen consumption over time of reaction.

### 3.5. Effect of Oxidizing Agent

When implementing a process on an industrial scale, it is necessary to use a cost-effective oxidizing agent. In the context of industrial oxidation processes, air may be a more suitable option compared to oxygen. Thus, the oxidation processes of 2-EHAL with oxygen and air were compared. The results are depicted in Table 5.

Replacing oxygen with air led to a significant reduction in 2-EHAL conversion and the 2-EHA selectivity (entry 1 and 2). Additionally, increasing the reaction temperature when using air did not yield the desired effect of (entry 3 and 4). The obtained data indicate that utilizing air would require higher pressure.

## 4. Conclusions

(1) The study demonstrated the feasibility of the oxidation process of 2-EHAL to acid under mild conditions with oxygen in the presence of NHPI as a catalyst in i-BuOH as a solvent.
(2) 2-EHA was obtained with high selectivity of 99.4% and a conversion of 59.0% (30 °C, 3 h, 0.1 MPa, 5 mol% NHPI, i-BuOH)
(3) The developed method holds potential for implementation in industry due to its high selectivity, cost-effective oxidizing agent, and mild reaction conditions.
(4) i-BuOH enables the dissolution of NHPI in the reaction mixture, does not undergo esterification under the reaction conditions, and facilitates heat exchange. Additionally, the use of i-BuOH as a solvent provides an opportunity for potential producers of 2-EHAL from butanal to utilize this less valuable alcohol.
(5) It was observed that the use of air is feasible, however, it would require higher pressure.

## Figures and Tables

**Table 1:** The influence of the type of solvent on the oxidation of 2-EHAL.

Entry	Solvent	Conv. 2-EHAL [%]	Sel. 2-EHA [%]	Sel. Heptane [%]	Sel. 3H=O [%]	Sel. 3HFE [%]	Sel. 3H-OL [%]
1	AcOH	99.9	61.9	1.6	nd	8.5	6.1
2	MeCN	99.8	47.1	nd	1.5	1.9	8.5
3	PhCH_3_	99.5	60.7	0.7	0.5	21.7	0.5
4	Heptane	98.9	68.7	**	0.4	14.4	2.2
5	Decane	98.8	71.5	0.8	0.2	16.0	1.0
6	2-ethylhexanol	55.2	93.0	1.6	2.1	0.8	6.3
7	i-BuOH	47.8	92.6	nd	0.9	0.8	1.0
8	n-butanol	42.7	97.0	nd	*	0.7	4.1
9	MeOH	0.0	nd	nd	nd	nd	nd

2-EHAL 2 mmol, solvent 8 cm^3^, NHPI 5% mol, 30 °C, 3 h, 800 RPM, oxygen—0.1 MPa, “nd” not detected, - * the solvent has the same retention time as 3H=O, ** solvent.

**Table 2:** The influence of the amount of solvent on the oxidation of 2-EHAL.

Entry	Volume of Solvent (cm^3^)	Conc. of 2-EHAL (mol/dm^3^)	Conv. 2-EHAL (%)	Sel. 2-EHA (%)	Sel. Heptane (%)	Sel. 3H=O (%)	Sel. 3HFE (%)	Sel. 3H-OL (%)
1	8	4	47.8	92.6	nd	0.9	0.8	1.0
2	6	3	59.1	95.5	nd	1.0	1.0	0.7
3	4	2	61.3	94.9	nd	0.7	1.1	0.1
4	2	1	59.0	99.4	nd	0.6	1.4	0.2

2-EHAL 2 mmol, solvent i-BuOH, NHPI 5% mol, 30 °C, 3 h, 800 RPM, oxygen—0.1 MPa, “nd” not detected.

**Table 3:** The influence of the amount of NHPI on the oxidation of 2-EHAL.

Entry	NHPI (mol%)	Conv. 2-EHAL (%)	Sel. 2-EHA (%)	Sel. Heptane (%)	Sel. 3H=O (%)	Sel. 3HFE (%)	Sel. 3H-OL (%)
1	8	61.2	96.5	nd	0.2	1.5	nd
2	6	62.1	95.2	nd	0.3	1.5	nd
3	5	59.0	99.4	nd	0.6	1.4	0.2
4	4	55.8	94.4	nd	0.2	1.4	nd
5	2	47.7	96.8	nd	0.3	1.5	nd
6	-	22.9	23.4	nd	nd	0.2	nd

2-EHAL 2 mmol, solvent 2 cm^3^ i-BuOH, 30 °C, 3 h, 800 RPM, oxygen—0.1 MPa, “nd” not detected.

**Table 4:** The influence of temperature and time on the oxidation of 2-EHAL.

Entry	Temp. (°C)	Time (h)	Conv. 2-EHAL (%)	Sel. 2-EHA (%)	Sel. Heptane (%)	Sel. 3H=O (%)	Sel. 3HFE (%)	Sel. 3H-OL (%)
1	30	3	59.0	99.4	nd	0.6	1.4	0.2
2	35	3	70.3	75.0	nd	0.1	1.2	0.0
3	40	3	73.9	85.2	nd	0.4	1.6	0.1
4	50	3	76.0	71.4	nd	0.6	1.9	0.4
5	60	3	99.9	59.3	0.1	0.3	1.8	0.8
6	35	0.5	59.7	76.2	nd	0.2	1.1	0.2
7	35	1	62.8	63.0	nd	0.1	0.6	0.0
8	35	2	69.2	71.0	nd	0.1	1.1	0.0
9	35	3	70.3	75.0	nd	0.1	1.2	0.0

2-EHAL 2 mmol, solvent 2 cm^3^ i-BuOH, NHPI 5% mol, 800 RPM, “nd” not detected.

**Table 5:** Effect of the oxidizing agent and temperature on 2-EHAL oxidation.

Entry	Temp. (°C)	Oxidizing Agent	Conv. 2-EHAL (%)	Sel. 2-EHA (%)	Sel. Heptane (%)	Sel. 3H=O (%)	Sel. 3HFE (%)	Sel. 3H-OL (%)
1	30	oxygen	59.0	99.4	nd	0.6	1.4	0.2
2	30	air	48.0	86.6	nd	1.1	1.3	0.5
3	40	oxygen	73.9	85.2	nd	0.4	1.6	0.1
4	40	air	58.1	63.4	0.1	0.4	1.0	0.5

2-EHAL 2 mmol, solvent 2 cm^3^ i-BuOH, NHPI 5% mol, 30 °C, 3 h, 800 RPM, “nd” not detected.

## Data Availability

All data are available in the manuscript or upon request to the corresponding author.
